# Asepsis and the Sterilization of Instruments

**Published:** 1903-05

**Authors:** Samuel A. Hopkins

**Affiliations:** Boston, Mass.


### ASEPSIS AND THE STERILIZATION OF INSTRUMENTS.[1]

BY SAMUEL A. HOPKINS, M.D., D.D.S., BOSTON, MASS.

To practise aseptic dentistry is impracticable, if not impossible, but to sterilize instruments after using requires no especial knowledge or skill. It does, however, call for moral backbone and for character of the highest type.

The practice of dentistry has always called for the exercise of extraordinary strength of character and honesty of purpose, for the patient is almost at the mercy of the operator and can have but the slightest understanding of how well or how badly he does his work. A plausible liar can fool his patients for years, but he doesn't like to meet members of his own profession, and generally stays away from dental society meetings, lest his dishonesty be discovered.

A high moral standard has always been an essential to dental practice, but never has the advice of Polonius, "To thine own self be true," been more applicable to the dental profession than since the advent of bacteriology made the sterilization of instruments a responsibility from which we have no right to shrink.

We know—have known for years—all about the sterilization of instruments.

We know, and have known for years, of the possibility of transmitting the germs of syphilis, of tuberculosis, and of other diseases by means of unclean instruments. We have known of the ease with which pus-producing forms can be carried on the mouth-mirror and instruments, and by the hands.

We know that the cotton with which we have wiped out an abscess or which we have taken out of a suppurating tooth is often left on the table or falls upon the floor, and we know that in its meshes are perhaps millions of staphylococci, and perhaps streptococci, capable of giving rise, under suitable conditions, to abscesses of a serious nature.

We need not be told that the mouth-mirror in our examining-room should be sterilized whenever it comes out of a patient's mouth and before it is used for the next examination. Our con-

---

[1] Read before the Harvard Odontological Society, November 20, 1902, and before the American Academy of Dental Science, February 4, 1903.

science pricks us every time we rinse it off and wipe it, and say, "I guess that's clean," for we know that we have taken a chance of introducing some germ into the patient's mouth that was not there before.

All this knowledge has become so familiar that we deem it almost commonplace.

Most of us long ago accepted the germ theory of disease. We have firm faith in the doctrine that many diseases are occasioned by living micro-organisms that usually find their way into the body through the mouth.

Even the most rigid and sceptical homœopathist (whose principles we respect, though we may not accept them), even the most ardent Christian Science crank, prefers aseptic surgery to a surgical operation performed amid filthy surroundings and with unclean instruments. And this preference he can scarcely show without admitting that bacteria are the exciters of disease.

How comes it, then, that a large body of educated men, many of whom have received special instruction in bacteriology, should be careless in their application of asepsis to their professional work? How is it that a body of men who probably put forth more conscientious, painstaking labor in the course of the average day than any class of men in the community should fail to include in their high moral code a practical plan for the sterilization of instruments?

How is it possible for a man with well-developed moral fibre, such as most dentists possess, who is honest in every detail of his work, who is constantly sacrificing his own convenience and giving his strength and health for the good of mankind,—how is it possible, I say, for such a man, knowing what bacteria are, how they may be transmitted, and what evils they may produce, and knowing, too, how to prevent these evil results,—how is it possible for any dentist to neglect any precaution that will protect his patient from the remotest possibility of infection?

It does seem strange that there should be a single dentist left who does not take the precaution necessary to protect absolutely his patients against contamination, but I am afraid we must admit that many of us are guilty of occasional sins of omission. You have only to watch a clinic at any dental society meeting to see an illustration of my meaning. Usually at such meetings there are no wash-bowls provided where a man may wash his hands. Mouth-mirrors are carried in the waistcoat pocket, and are thrust from one mouth into another with no attempt at sterilization and only an attempt at ordinary cleanliness.

The hands of the seeker after truth are thrust first into a mouth where a clever piece of bridge-work is on exhibition, and with only the most rudimentary attempt at cleanliness proceed to examine a mouth afflicted by cancer.

The fact that the sterilization of instruments has not become general among dentists is, I think, pretty clearly shown by the fact that the most important dental societies in the State, and in the country, when holding conventions and giving clinics, fail to provide means for even the ordinary cleansing of instruments, and leave the clinicians to depend on such antiseptics as they may have at hand.

Do not misunderstand me. I am not here to berate the profession nor to bring accusations of neglect of duty. I am trying to picture the condition of affairs as I see it to-day in many schools, in many offices, and in the public clinics given by our dental societies. Let us be honest with ourselves, and admit that the picture is not greatly overdrawn, and let us seek the explanation. The average dentist is an eminently practical person. Show him a way by which he can improve the condition of his patient's mouth, and no consideration of self and no thought of the trouble involved will prevent his adopting that method. He is so intensely practical that there is danger of his becoming empirical.

In the matter of asepsis, the busy dentist has many excuses for not being greatly impressed by the necessity for sterilizing his instruments, and when all things are considered, it must be confessed that the number of known cases where infection has been carried by dental instruments is comparatively small. We know that, so far as septic infection is concerned, the mouth, owing to its enormous blood-supply and the abundance of leucocytes in the tissues, is more resistant than any other part of the body. This is not due, as many suppose, to any antiseptic property contained in the saliva itself. There may, and probably do, exist in the mouth secretions many non-pathogenic bacteria that excite the leucocytes to action, and in this way protect wounds from pathogenic or pyogenic micro-organisms. This probably explains why animals lick their wounds. Human saliva, when sterilized, not only has little retarding action on bacteria, but acts as a very good culture medium for many forms. If it has any power to protect against the inroads of disease, that power lies in the non-pathogenic bacteria which it contains.

We have been accustomed for generations to perform, and to see performed, operations in the mouth which, if undertaken in any other part of the body without aseptic precautions, would result in abscess, septicæmia, or lock-jaw.

Suppose you made such a wound in the foot, leg, or arm as is ordinarily made in the mouth by the extraction of three or four teeth, what would become of the patient if it were allowed to fill up with all sorts of foreign matter and remained untreated? It would naturally result in an unprecedented boom in cemetery lots. Yet we see these great excavations in the mouth, ragged edges of gum tissue, fractured alveolar process, all caused by the extraction of a tooth, and all healing without the slightest treatment or the least suggestion of cautionary measures. Food particles fill up the wound; armies of bacteria flourish, multiply, and die; all the fluids of the mouth find their way in and assist in maintaining an active process of fermentation or putrefaction; and yet, even in the filthiest mouth, after extraction by the filthiest dentist using the filthiest instruments, we are bound to confess that serious results seldom follow such operations.

I am not pleading for lack of cleanliness, but I wish to be fair, and to show why dentistry has not pinned its faith to aseptic operations, as surgery is obliged to do. We have not felt the necessity, and we are practical enough to know that the risk isn't really very great if we don't strictly sterilize our instruments after each patient.

We know too, even if it has not been carefully explained to us, that the great safeguard against bacteria, the impenetrable wall against which the germs of disease may dash themselves in vain, is sound, healthy, and unbroken epithelial tissue. Whether it be in the mouth or upon the surface of the body, healthy epithelial tissue forms an impassible barrier to all forms of bacteria. We know, however, that while this is a safeguard to be trusted, it is not to be fooled with, for we know that blood-poisoning frequently follows a wound too small to have been noticed, nor can we ever be sure that in the mouth the epithelial covering is not broken or congested and ready for the admission of bacteria.

Surgeons have been known, time and time again, in the frenzy of their devotion to the scrubbing-brush, to so scrub away and irritate the epithelium of their hands and arms as to invite the entrance of bacteria, and many septic fingers and arms, as well as general and fatal poisoning, have followed this vigorous but ill-judged adherence to the principles of aseptic surgery. This has resulted in the use of rubber gloves among surgeons to a certain extent. There are certain advantages and certain disadvantages in the use of the rubber gloves by surgeons, but for the dentist the gloves are hardly to be thought of except in dealing with a syphilitic or other infectious case. On the whole, we have reason, I think, for believing that in view of the vast number of dental operations performed, the evidence that can be gathered would hardly show any great number of cases wherein disease has been carried by dental instruments, and it is for this reason, and for the other reasons I have alluded to, that the practical dentist has not shown an active interest in the lessons bacteriology teaches, and has failed to seize with avidity the idea of absolute protection to his patient by means of sterilized implements of practice.

Nevertheless, the idea demands our respect, and the knowledge we have acquired in the past twenty years in relation to the transmission of disease by bacteria places upon us a responsibility so grave, so sacred, that to neglect it is scarcely less reprehensible than to become a contributing party in a case of manslaughter.

Do not let us shirk this responsibility, but let us face it with the will and power which have characterized all advances in this profession. You know, and I know, that the fact that infection by means of dental instruments is unusual should not enter into this question at all. In England, where chloroform is used as an anæsthetic, it has been estimated that one life is lost in every five thousand cases operated on. We can conceive of circumstances where, knowing this danger, it would be good judgment to use chloroform, as, for instance, in operating at night, when the danger of setting fire to ether must be considered, or in cases where the retching and vomiting following ether would endanger the success of the operation. We can easily justify such a risk and continue to confide in the judgment of the surgeon, in spite of fatal consequences. On the other hand, suppose the risk of fatal consequences in using an unclean—and by that I mean an unsterilized—knife was only one in ten thousand; I am not sure that the actual risk is greater; but would any one condone a surgeon's action in using such an instrument, when boiling water and other means of sterilization are to be had for the asking? Have we not a right, in view of our present knowledge of bacteriology, to regard the surgeon guilty of manslaughter who contributes to the death of a patient by using an unsterilized knife? Let us bring this home to ourselves. Can we ever be sure, in using an unsterilized instrument, that we are not conveying a disease germ to our patient's mouth, and can we be sure that the germ so conveyed will not find the conditions ripe for its development and for the propagation of disease?

I have recently completed some experiments upon the micrococcus tetragenus, the staphylococcus pyogenes aureus, and the micrococcus lanceolatus or croupous pneumonia germ, which show beyond question that these forms vary greatly in their virulence in different mouths. These experiments bear on the question of sterilizing instruments in this way: It can be fairly inferred that a bacterium which is quite inactive and harmless in one mouth might, when transferred to another, find conditions which would cause it to become exceedingly virulent and a menace to health. Moreover, the dental profession has, with few dissenting voices, accepted Miller's theory of the bacterial origin of caries. At present we do not attribute the formation of lactic acid to a particular micro-organism. I have, myself, isolated sixteen different forms from the mouth, capable, under favorable conditions, of producing this acid, and it is said that there are not less than a hundred forms of bacteria in existence that have this interesting property. We cannot tell as yet what forms would be active in one mouth and what would be active in another, but we are pretty sure that in excavating a carious tooth preparatory to filling, our instrument has come in contact with some form that, transferred to another mouth, might give rise to caries.

Are we then justified in taking the risk of introducing that micro-organism into the mouth of our next patient by a failure to sterilize that instrument?

Besides the common mouth forms, besides the lactic acid producing forms, we know that the germs of nearly all diseases enter the system through the mouth. They come with food, water, air, and from the fingers thrust into the mouth.

I fancy that it is true that disease germs are generally harmless, and I mean by that it is probable that most of us have again and again in our lives taken in the germs of typhoid, or some other serious disease, and yet, because the system was not in a condition to be affected, or because the culture material was not right for its development, it died out without producing the disease. Yet in spite of this immunity (upon which we can only speculate, since we have no way of determining), we decline to receive disease germs into the system if we can by any reasonable means avoid them, and if the sterilization of dental instruments will in any way lessen the possibility of transmitting disease, we have no longer an excuse for declining to take this trouble.

Now, as to how to sterilize the instruments. The methods are simple and familiar. If we once accept the responsibility and go about it with the determination to do our duty, there will be no difficulty about ways and means. You cannot improve much over boiling in water to which a pinch of washing soda has been added to prevent rusting. Fifteen minutes will destroy any bacterium likely to be found in the mouth. It will not, however, always destroy spores, which are extremely resistant to heat and to chemical agents. Still, I think we may ignore spores, for two reasons: in the first place spores are only developed on old culture media after the chief ingredients which nourish the microscopic plants have been exhausted from the soil, and in the mouth we have few such conditions. The soil or culture medium in the mouth is constantly being renewed, and there is unceasing activity of growth which precludes the likelihood of spores being developed. Secondly, the coccus forms which predominate in the mouth do not produce spores. If spores develop, it will generally be because the instrument has been laid away for several days before sterilizing.

Boiling water will effectually sterilize dental instruments. There are, however, many dental implements which cannot, without injury, be submitted to boiling water, but these can be sterilized quickly and effectually in a formalin oven. The Schering formalin sterilizer and lamp are probably familiar to you all. If you wish me to be accurate, I will say that in experiments which I have made, instruments kept in the oven for ten minutes, with three small or one large pastile burning, showed no growth at the end of twenty-four hours, when placed in bouillon tubes; a slight growth was noticed at the end of forty-eight hours. Similar instruments not sterilized showed distinct growth in twelve hours, and profuse growth of many varieties in twenty-four hours. Instruments kept in the oven for fifteen minutes showed no growth. Fifteen minutes may therefore be considered effectual.

One thing is to be said about the use of formalin: the instruments should be both clean and dry before being put in the oven. By clean, I mean that there should be no dried blood, bits of carious tooth, or other unclean substances on the instruments, since the formalin gas will not penetrate deeply nor will it prove effective on wet surfaces. If you use formalin, the best way to manage is to have the formalin oven in your office. You have a perfect right to inspire confidence in your patient by doing the sterilizing under his eyes. It is a good way to make public opinion. When our patients refuse to patronize a man who is untidy, and who does not sterilize his instruments, the reform in dental practice will come quickly. When you have dismissed a patient, give the instruments to a trusty servant to wash and wipe dry, and then put them into the sterilizer. It is better not to leave the lamp burning longer than is necessary to convert the pastile into gas, as the heat generates moisture, and there will be a sweating process which will rust the instruments and the oven as well. Often this is unavoidable, and there is a deposit on the instruments resembling dust, which must be wiped off with a sterile napkin.

It is a good plan to have your boiling water also in your office. A shelf can be constructed sufficiently high, so as to be out of the way of small children, and a Bunsen burner, connected with the neighboring gas-jet, a lamp, or an electric heater, as the case may be, placed on this shelf, and over this a pot of boiling water may stand all day, and the instruments be placed in it. It is better, for convenience in handling, to have a wire basket with a solid bottom, and to place the instruments in this. The basket can be placed in the boiling water and removed with little inconvenience and no danger of burning the fingers. It is more convenient to have both methods at hand, and there is scarcely any expense attached to either. The office itself should be well sterilized from time to time, and this may be done by burning formalin. You close the windows tightly, pull out the drawers of your instrument case, put a half-dozen pastiles in the cup of the lamp, light the lamp, close the door, plug up the key-hole, and leave it until the following morning. You will find an odor of formalin, and your eyes and nose will tingle when you first enter the room, but five minutes with an open window will remove the odor, and the place will be wholesome, sweet, and sterile. I have tried any number of experiments to prove the efficacy of this method of sterilization, and I can assure you it is most effective for superficial sterilization. At the Medical School I had a room to work in that contained about eight hundred cubic feet of space. Three or four fresh formalin pastiles would render the place so sterile that the dust on the shelves (and the place was covered with dust) could be brushed into a tube of bouillon and the tube set in the incubator without any growth showing itself.

The need for general office sterilization arises because the fingers in picking up an instrument from the cabinet or tray may come in contact with other instruments, things will drop on the floor, and the air of the room is likely to become contaminated. You are sure of clean instruments and clean air if you sterilize your room every night, but you must always remember that formalin has not the power of penetration that it was once supposed to have, and must be looked upon as a surface sterilizer. For this reason the instruments should be washed and dried first. This is especially true of burs and files. The hands cannot be rendered absolutely sterile without an expenditure of time and trouble which is out of all proportion to the necessities of the case, but they may be rendered practically sterile by the use of permanganate of potash, or, better still, by a new preparation called Sublamine.

It is an ethylenediamine-sulphate of mercury, about as powerful as the corrosive sublimate, but does not irritate the hands. It dissolves well in water, can be used with soapsuds, which is not true of the sublimate, penetrates deeper, and leaves the hands smooth. After infective material has touched the hands they should always be washed in sublamine. We may as well give up the idea of strictly aseptic dental operations, because we cannot possibly sterilize the mouth, and the moment an instrument enters it becomes contaminated.

The public, however, has a right to expect security against the mouth bacteria of others. Our patients are only reasonable in demanding that they be not exposed to the germs contained in the mouth of the patient that preceded them, and it is our business to insure them this immunity, but to practise aseptic mouth dentistry or surgery is often beyond the limit of our skill. I know of no method of destroying the bacteria in the mouth that will not also destroy the patient. I say this soberly after years of experimentation. I continue to use antiseptics in all cases where suppuration occurs, and am convinced that they do an immense amount of good, but I have never been able to keep a mouth in a strictly aseptic condition for ten minutes at a time, and for our purposes it is not necessary, nor am I sure that it is altogether desirable, that the mouth should ever be sterile. For the ordinary operations in dentistry our responsibility ends when we have given a definite assurance that we are not conveying to the mouth of any patient a bacterium that was not there before. For surgery and for extractions we will naturally take all the precautions possible to lessen the danger of infection from germs that exist in the patient's own mouth, and here we may gain much from the use of suitable antiseptics.

I am proud to be able to say, in closing, that in both of the dental schools in Boston effective measures are taken for the sterilization of instruments, and every boy in these schools has impressed upon him the necessity for thoroughness in this particular.

As soon as the necessity is generally appreciated public opinion will demand, in no uncertain voice, protection at the hands of the dentist, and the laggard dentist who is content to take his chances and run for luck will find himself supplanted by these young men from our dental schools, who understand their moral responsibility and have backbone and determination enough to practise with clean implements, clean hands, and a clean conscience.

